# Age- and Sex-Specific Reference Intervals for Amino Acids and Acylcarnitines by Tandem Mass Spectrometry in a Kazakhstani Paediatric Population: An Indirect Data-Mining Study

**DOI:** 10.3390/diagnostics16172766

**Published:** 2026-08-28

**Authors:** Yerlan Suleimenov, Ayat Assemov, Dana Rsaldina, Amir Baikatov, Albina Omarova, Antonina Bespalko

**Affiliations:** 1 Head Office, CDL Olymp LLP (Laboratory Network), Astana 010000, Kazakhstan; yerlan.suleimenov@kdlolymp.kz (Y.S.); albina.omarova0604@gmail.com (A.O.); a.bespalko@kdlolymp.kz (A.B.); 2 School of Medicine, Nazarbayev University, Astana 010000, Kazakhstan; 3 R&D Department, MYRZA-KHAN LLP, Astana 010000, Kazakhstan; d.rsaldina@kdlolymp.kz; 4 Brain Institute, Farabi National University, Almaty 050040, Kazakhstan; amir.sapar@zoho.com

**Keywords:** reference values, tandem mass spectrometry, amino acids, acylcarnitines, newborn screening, dried blood spots, inborn errors of metabolism, indirect method, paediatrics

## Abstract

**Background/Objectives:** Interpretation of amino-acid and acylcarnitine results in newborn screening depends on population-appropriate reference intervals (RIs), and no locally derived paediatric RIs have been published for the general Kazakhstan population. We aimed to establish the first age- and sex-specific RIs for a tandem mass spectrometry (MS/MS) panel measured from dried blood spots (DBSs) by flow injection analysis tandem mass spectrometry (FIA-MS/MS) in a Kazakhstani paediatric population using an indirect (data-mining) approach. **Methods:** All amino-acid and acylcarnitine results (1 January 2022 to the data freeze on 20 July 2026) were extracted from the consolidated laboratory information system of a 21-branch network; after cleaning, deduplication and selection of conditionally healthy subjects, RIs (2.5th, 50th, 97.5th percentiles with 90% bootstrap confidence intervals) were computed per CLSI EP28-A3c across CALIPER age strata, with the neonatal period split into 0–7/8–14/15–28-day strata, and cross-validated by the canonical refineR package run without flag-based selection, the CLIR/Mayo database and a comparable Turkish study. **Results:** From 687,726 results in 4198 patients, the conditionally healthy cohort comprised 3589 subjects; the large majority of analyte–age strata permitted full CLSI EP28 computation. Canonical refineR and flag-based estimates agreed within ±30% in 91% of key-marker limits (median relative difference 9.6%), indicating that flag-based selection did not materially bias the upper limits. Reference intervals are reported for all amino acids and all 36 acylcarnitines of the panel. The valine 97.5th-percentile upper limit exceeded its 250 µmol/L decision limit at 0–28 days (275.3 µmol/L). **Conclusions:** We provide locally derived and internally cross-checked paediatric MS/MS RIs, pending prospective verification, and flag two actionable findings: inter-site harmonisation of several key markers and the overlap of the upper-normal valine limit with its clinical decision limit.

## 1. Introduction

Tandem mass spectrometry (MS/MS) measurement of amino acids and acylcarnitines from dried blood spots (DBSs), performed by flow injection analysis tandem mass spectrometry (FIA-MS/MS), underpins the laboratory diagnosis of inherited metabolic disorders and forms the analytical core of expanded newborn screening programmes [1,2]. The clinical value of every measured analyte depends on the reference interval (RI) against which it is interpreted, because both the recognition of disease and the avoidance of false alarms hinge on where the upper and lower decision limits are placed [3,4]. Throughout this paper, we distinguish three concepts that are easily conflated: a population *reference interval* (the central 95% of the healthy distribution, the subject of this study); a *screening cut-off* (an action threshold tuned for sensitivity, e.g., the CLIR/Mayo post-analytical ranges); and a *clinical decision limit* (a diagnostic threshold). These three concepts are not interchangeable.

Reference intervals vary with the analytical platform, the population and pre-analytical conditions, and authoritative guidance therefore recommends that each laboratory verify or establish its own intervals rather than transfer them uncritically [3,5,6]. This requirement is particularly acute in the neonatal period, when amino-acid and acylcarnitine concentrations change rapidly over the first weeks of life, so that a single neonatal interval may mask clinically important within-month dynamics. For paediatric and especially neonatal populations, the direct approach of recruiting large numbers of demonstrably healthy reference subjects is ethically and logistically difficult, which is why landmark initiatives such as CALIPER and indirect data-mining methods have become central to the field [7,8,9]. Indirect methods derive RIs from results already accumulated in the laboratory information system (LIS) of routine testing, exploiting the fact that the majority of tested individuals are not affected by the rare disorders the panel targets [10,11].

Despite this, no paediatric MS/MS reference intervals have been published for the general Kazakhstan population; existing national MS/MS work has focused on selective screening protocols rather than population reference intervals [12]. Laboratories in Kazakhstan therefore rely on imported, vendor-supplied or literature-derived limits whose applicability to the local population is unverified [5]. The aim of this study was to establish, for the first time, age- and sex-specific RIs for the amino-acid and acylcarnitine FIA-MS/MS panel used across a national, multi-branch laboratory network in Kazakhstan, following the CLSI EP28-A3c indirect method, and to cross-check these intervals using a second, methodologically independent estimator and external reference data.

## 2. Materials and Methods

### 2.1. Subjects

This was a retrospective study based on routine data extracted from the consolidated LIS (Microsoft SQL Server 2019; Microsoft Corporation, Redmond, WA, USA) of a laboratory network operating 21 branches across Kazakhstan. All results of the amino-acid and acylcarnitine MS/MS panel (internal test code B09.801.019) reported between 1 January 2022 and the data freeze on 20 July 2026 were retrieved. This study used only residual, routinely collected, fully de-identified LIS data, with no intervention and no contact with patients. Under the Code of the Republic of Kazakhstan No. 360-VI of 7 July 2020 “On the Health of the People and the Healthcare System” (Articles 227, Biomedical research, and 228, Bioethics commissions) and Order of the Minister of Health of the Republic of Kazakhstan No. KR DSM-310/2020 of 21 December 2020 “On approval of the rules for conducting biomedical research and the requirements for research centres”, this retrospective, non-interventional analysis of de-identified, routinely collected results does not constitute biomedical research requiring bioethical expertise; the assessment was additionally reviewed and confirmed in writing by the Local Bioethics Commission of CDL Olymp (reference OLC-0003, 16 July 2026). Written informed consent obtained from patients or their legal representatives at the sample-collection points explicitly covers the collection, processing and transfer of personal data for the purposes of scientific research and the formation of electronic information resources (institutional Orders on electronic informed consent Nos. KDL-691–698/MX-129 of 24 June 2025), so no separate study-specific consent was required, consistent with published guidance on consent and ethical approval in laboratory medicine [13]. Patients were linked across analytes by a non-identifying internal key.

A result was eligible for the conditionally healthy cohort if it carried no out-of-range flag for the analyte concerned, with one pre-specified exception: the two non-specific analytes glutamate and glutamine were exempted from the flag-based exclusion rule. In this dataset, glutamate flagged high in about 19% of records and glutamine flagged low in about 32%; such rates are incompatible with a genuinely abnormal subpopulation, so treating this over-flagging as pathology would have removed otherwise healthy children. The effect of the rule is quantified in Table 1. All reference-interval tables in this paper (main text and Supplementary Table S1) are computed on this single adopted cohort.

### 2.2. Methods

All measurements were produced on the network’s routine FIA-MS/MS platform, a SCIEX Triple Quad™ 4500 system (SCIEX, Framingham, MA, USA), using the NeoBase™ 2 Non-derivatised MSMS kit (Revvity, Turku, Finland); data were acquired with SCIEX Analyst software 1.7.2 (SCIEX, Framingham, MA, USA) and interpreted with ChemoView 2.0.4 (SCIEX, Framingham, MA, USA) software. All participating sites served as specimen-collection points; every dried blood spot (DBS) card was transported to a single central laboratory and analysed on one platform under standardised operating procedures, so there was no inter-site variation in instrumentation or analytical procedure. On receipt, cards were visually inspected and specimens rejected following the CLSI NBS01 guideline (its Section 5.7 and Appendix B [14]): insufficient, incompletely, or non-uniformly saturated, layered, clotted, contaminated, diluted, or inadequately dried spots, evidence of improper handling, transport, or storage, or incomplete or inconsistent identification; collection, drying, packaging, transport and storage followed CLSI NBS01 Chapter 6 [14]. Analysis used FIA-MS/MS on a SCIEX Triple Quad 4500 (SCIEX, Framingham, MA, USA) with the NeoBase 2 Non-derivatised MSMS kit (Revvity/Wallac Oy, Turku, Finland; No. 3044-0010). Instrument mass calibration, mass-accuracy verification and Q1/Q3 resolution were performed per the manufacturer using the PPGs Chemical Standards Kit (SCIEX, Framingham, MA, USA; No. 4406127) in positive and negative ion modes, with an additional reserpine MS/MS test (precursor m/z 609.3, product m/z 195.1); these instrument checks were distinct from the assay-specific calibration. Quantification used the stable-isotope-labelled internal standards supplied with the kit, with lot-specific internal-standard concentrations and relative response factors and no conventional external multi-point calibration curve. The same assay and standardised procedure were used throughout; different manufacturing lots were handled with lot-specific internal-standard and quality-control values and were never mixed. Internal quality control followed the manufacturer’s instructions: the kit Low and High DBS controls were analysed in duplicate on every plate and evaluated against the lot-specific target means and standard deviations and laboratory-established acceptable ranges, with total-ion chromatogram profiles and internal-standard signal intensities monitored throughout each run; results were accepted only when the pre-defined QC criteria were met; otherwise, the affected specimen or run was investigated and repeated. The laboratory also participates in external quality assessment through the CDC Newborn Screening Quality Assurance Program (NSQAP) and the ERNDIM Special Assays in Dried Blood Spots scheme, with satisfactory overall performance. Established MS/MS pre-analytical and analytical guidance was followed [1,15,16]. Raw result records were cleaned (removal of non-numeric, duplicate and technically invalid records) and deduplicated by retaining the earliest result per patient per analyte. Subjects were stratified into CALIPER age groups (0–28 days, 29 days–1 year, 1–4, 5–9, 10–13, 14–18 and over 18 years); to support first-week interpretation, the neonatal group was additionally split into 0–7, 8–14 and 15–28-day strata (and, where numbers allowed, 0–2 and 3–7 days) [7]. Age was computed at the date of registration (a proxy for sample collection), not at the result-reporting date, because the median collection-to-result interval of seven days would otherwise displace neonates out of the 0–28-day stratum.

### 2.3. Statistical Analysis

The “key markers” are defined as the nine principal amino-acid disease markers (phenylalanine, tyrosine, methionine, leucine, citrulline, valine, arginine, alanine, glycine) together with the primary acylcarnitines (C0, C3, C5, C8, C16, C18). For each analyte–age (and, where examined, analyte–age–sex) stratum, RIs were computed as the 2.5th, 50th and 97.5th non-parametric percentiles, with 90% confidence intervals of the limits estimated by non-parametric bootstrap (1000 iterations; random seed 42). The computation method was assigned by stratum size: full CLSI EP28-A3c (N≥120), robust (40≤N<120), descriptive (20≤N<40) and insufficient (N<20) [3]. Inter-site differences for the key markers were tested by the Kruskal–Wallis test with Bonferroni correction; sex differences were tested by the Mann–Whitney U test with Cohen’s d (biological-significance threshold |d|>0.3). Distributional assumptions were examined by formal normality testing, Box–Cox transformation analysis, Tukey outlier assessment, a 99th-percentile tail analysis relative to clinical decision limits and a rolling cumulative-sum (CUSUM) stability analysis. To test whether flag-based selection biased the intervals, every key–marker stratum was re-estimated from the full, unfiltered distribution using the published refineR package (canonical algorithm; R 4.6.1), with no flag-based selection [17]. Intervals were externally compared with the CLIR/Mayo Clinic post-analytical database [18,19,20] and with a comparable Turkish paediatric study [21]. Effect sizes were interpreted following Cohen [22]. Exact two-sided *p* values are reported to three decimal places; values below 0.001 are reported as p<0.001. Analyses used Python 3.14.6 (pandas, SciPy and matplotlib; Python Software Foundation, Wilmington, DE, USA) and R 4.6.1 (refineR; R Foundation for Statistical Computing, Vienna, Austria); all extraction and analysis code is deposited publicly (see Data Availability Statement). The analysis parameters and computational settings are summarised in Methods S1.

## 3. Results

### 3.1. Study Population and Data Sufficiency

**Figure 1:**
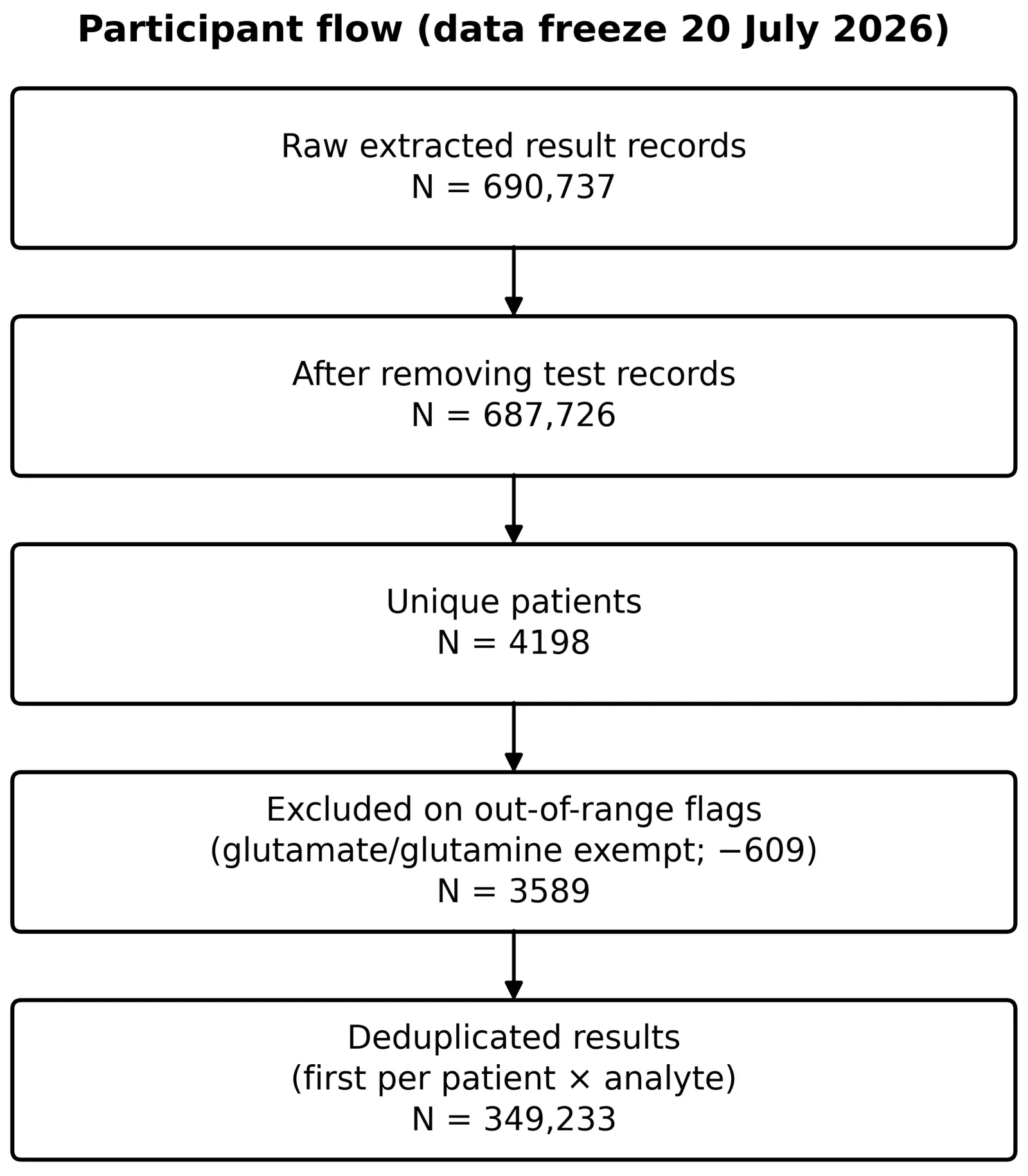
Participant flow from raw extraction to the deduplicated conditionally healthy cohort (data freeze 20 July 2026).

**Figure 2:**
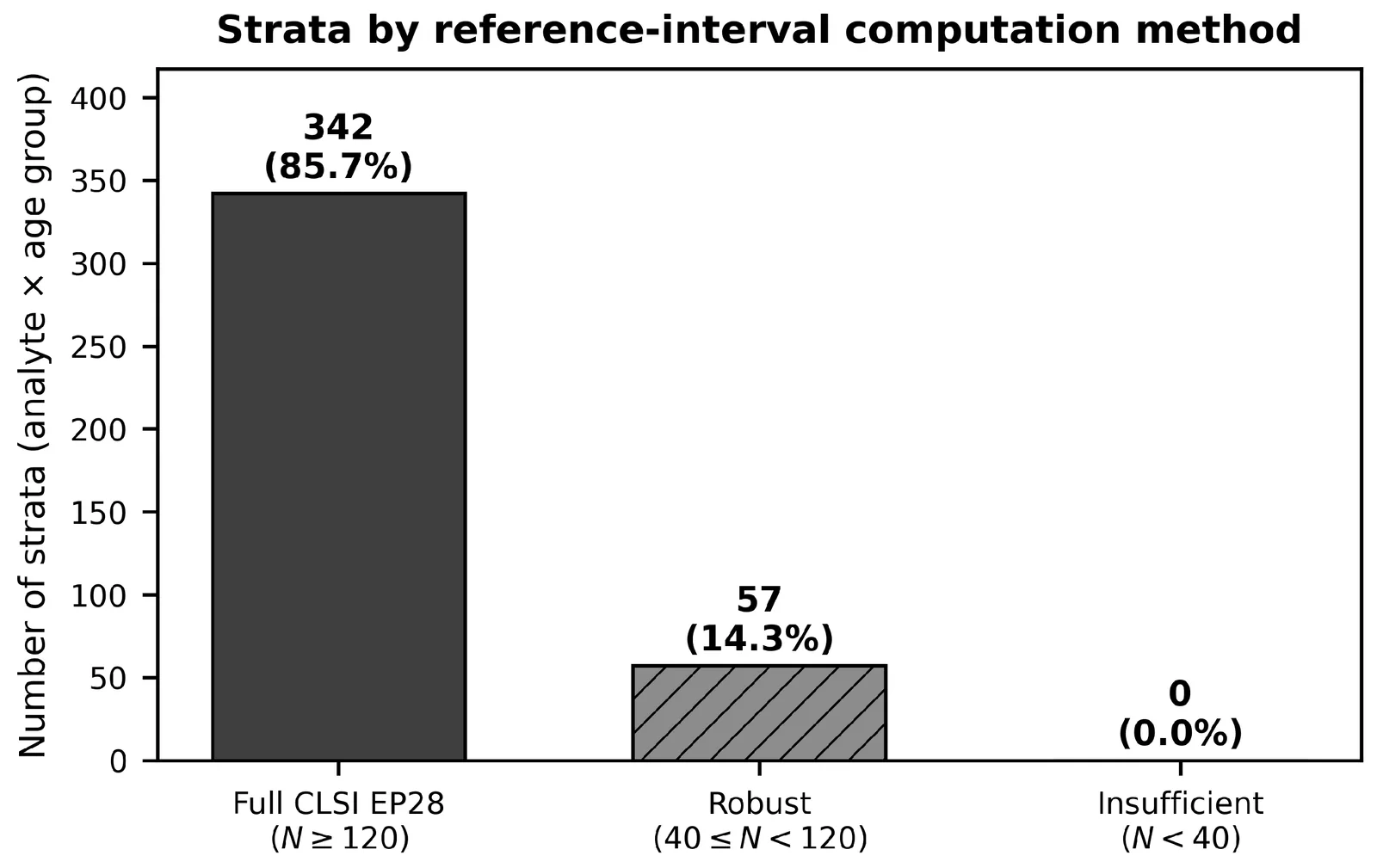
Data sufficiency of strata by computation method (399 quantitative-analyte strata; adopted cohort).

**Figure 3:**
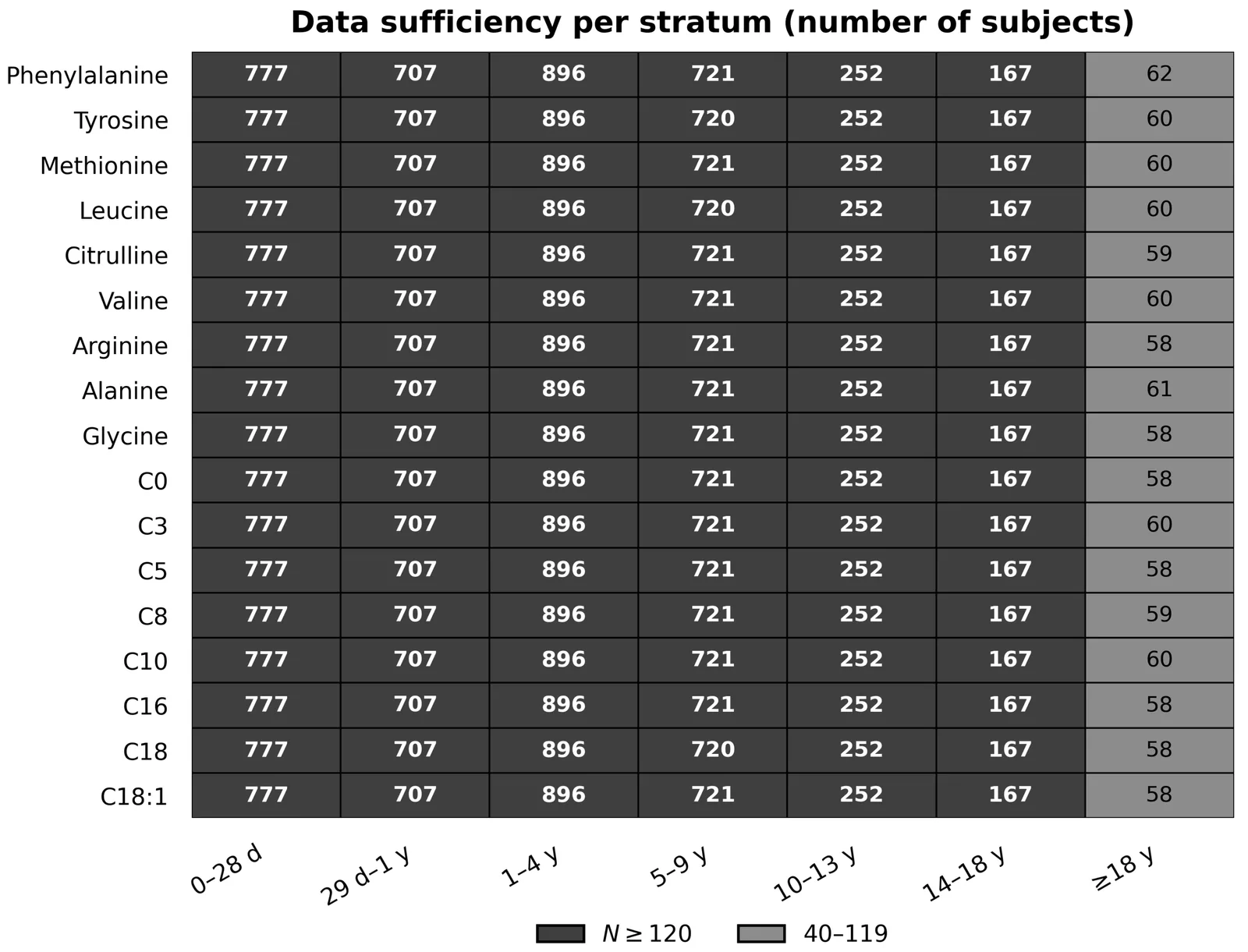
Per-analyte data sufficiency (*N*) across age strata for the key markers (adopted cohort).

**Figure 4:**
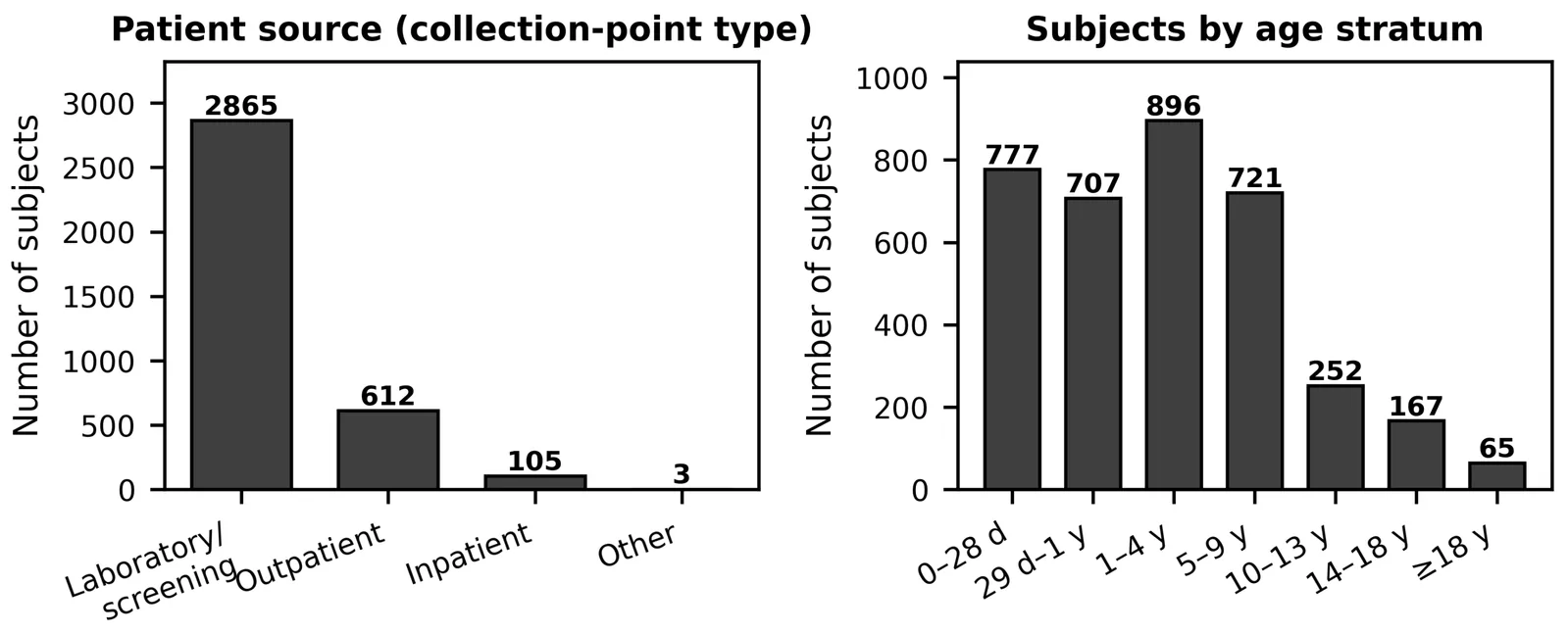
Patient source by collection-point type (**left**) and age distribution of the conditionally healthy cohort (**right**).

A total of 690,737 raw result records were retrieved; after cleaning, 687,726 results in 4198 unique patients remained (Figure 1). Exempting the two over-flagged non-specific analytes increased the conditionally healthy cohort from 1842 subjects under strict exclusion to 3589 (Table 1), yielding 349,233 deduplicated results. Of the 399 analyte–age strata for the quantitative analytes, 342 (86%) permitted full CLSI EP28 computation, 57 were provisional and none insufficient (Figure 2); data sufficiency was highest from the neonatal period to the 5–9-year group (Figure 3). The cohort was overwhelmingly an outpatient/screening population: by collection-point type, 2865 patients (80%) were registered through laboratory/screening points, 612 (17%) through outpatient collection rooms and 105 (3%) through inpatient/hospital sites (Figure 4). Neonates (0–28 days) accounted for 777 patients (21.7%) and subjects older than 18 years for only 65 (1.8%), consistent with the newborn-screening focus.

### 3.2. Reference Intervals for Key Markers

**Figure 5:**
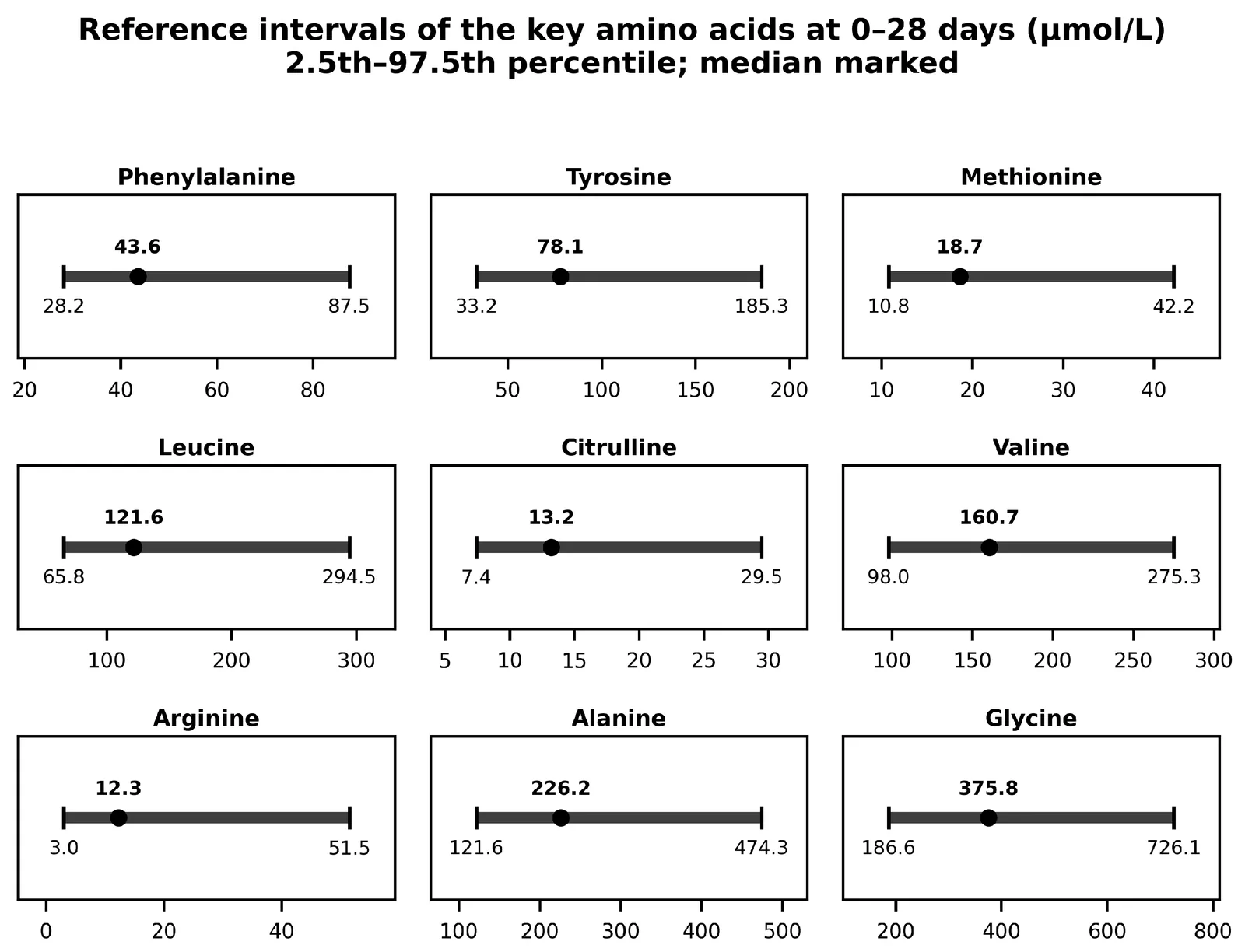
Neonatal (0–28 d) reference intervals of the nine key amino acids (bar, 2.5th–97.5th percentile; dot, median).

**Figure 6:**
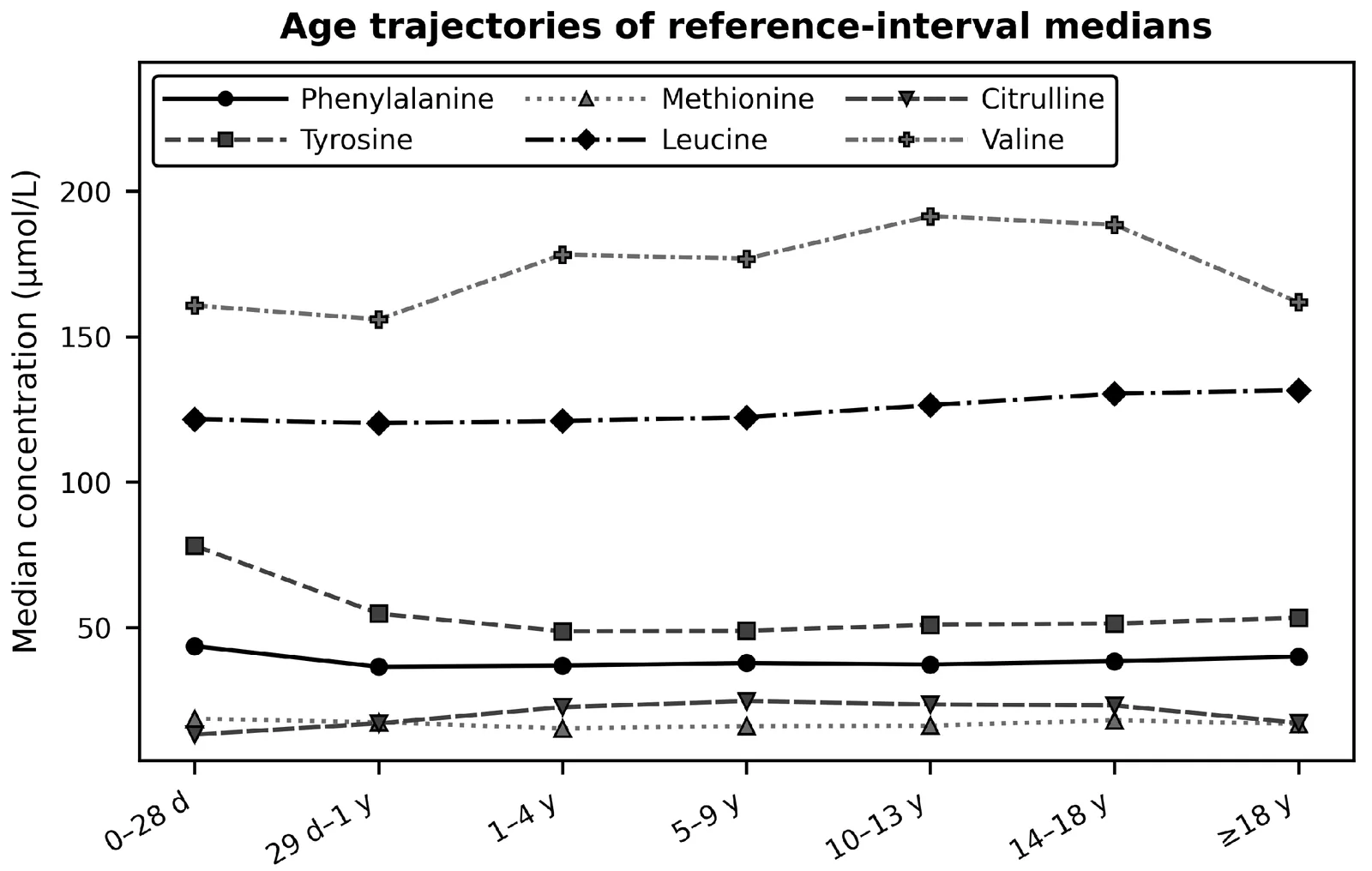
Age trajectories of reference-interval medians for key markers (adopted cohort).

Age-specific RIs for nine principal amino-acid markers are presented in Table 2; the full per-stratum table for all analytes (including acylcarnitines) is provided as Supplementary Table S1. Age trajectories were physiologically consistent, e.g., citrulline rose from a median of 13.2 μmol/L (0–28 days) to 22.6 μmol/L (1–4 years), and glycine declined with age. Neonatal distributions of the nine key markers are shown in Figure 5 and age trajectories of the medians in Figure 6.

### 3.3. Acylcarnitine Reference Intervals

Reference intervals were established for all 36 acylcarnitines of the panel across every age stratum (full set in Supplementary Table S1). The principal acylcarnitines used in screening are summarised in Table 3: free carnitine (C0), acetylcarnitine (C2), propionylcarnitine (C3), isovalerylcarnitine (C5), octanoylcarnitine (C8), decanoylcarnitine (C10), myristoylcarnitine (C14), palmitoylcarnitine (C16), stearoylcarnitine (C18) and oleoylcarnitine (C18:1). Neonatal medians and upper limits exceed those of later infancy for the long-chain species, consistent with the physiological acylcarnitine profile of the first weeks of life.

### 3.4. Fractional Neonatal Strata

**Figure 7:**
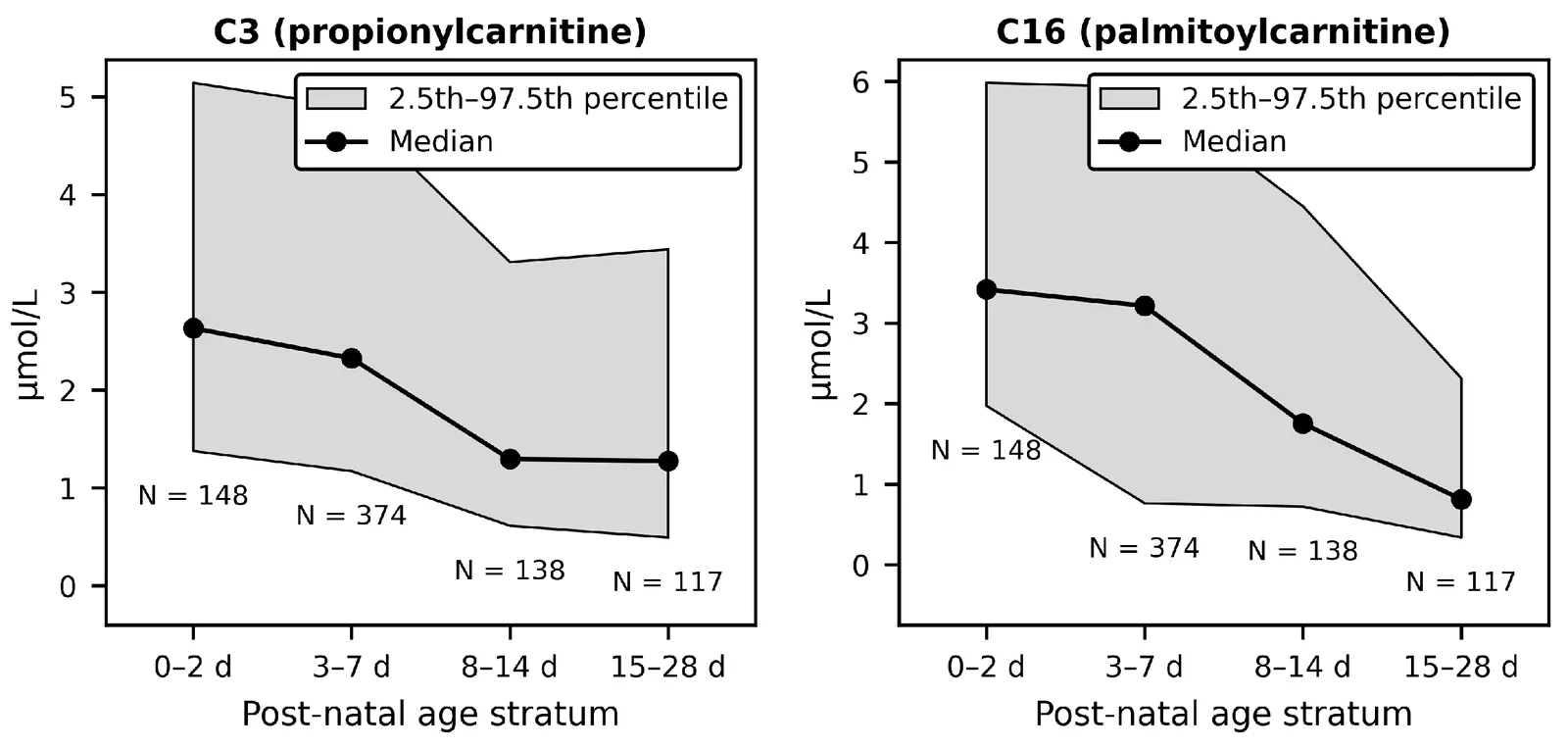
Dynamics of C3 (propionylcarnitine) and C16 (palmitoylcarnitine) across fractional neonatal strata (median and 2.5th–97.5th percentile band); *N* per sub-stratum annotated.

Splitting the first month into 0–7, 8–14 and 15–28-day strata revealed marked within-month dynamics that a single 0–28-day interval would obscure (Figure 7). Propionylcarnitine (C3) and palmitoylcarnitine (C16) both fell steeply over the first month: C3 from a median of 2.45 to 1.28 μmol/L and C16 from 3.29 to 0.81 μmol/L between the 0–7 and 15–28-day strata (Table 4). With the adopted cohort, the first-week stratum is well populated (e.g., C3 and C16: N=522 at 0–7 days, supporting full CLSI EP28 computation); strata with 40≤N<120 are marked provisional and those with N<40 insufficient (to be interpreted against international neonatal ranges).

### 3.5. Effect of the Selection Rule: Glutamate and Glutamine

**Figure 8:**
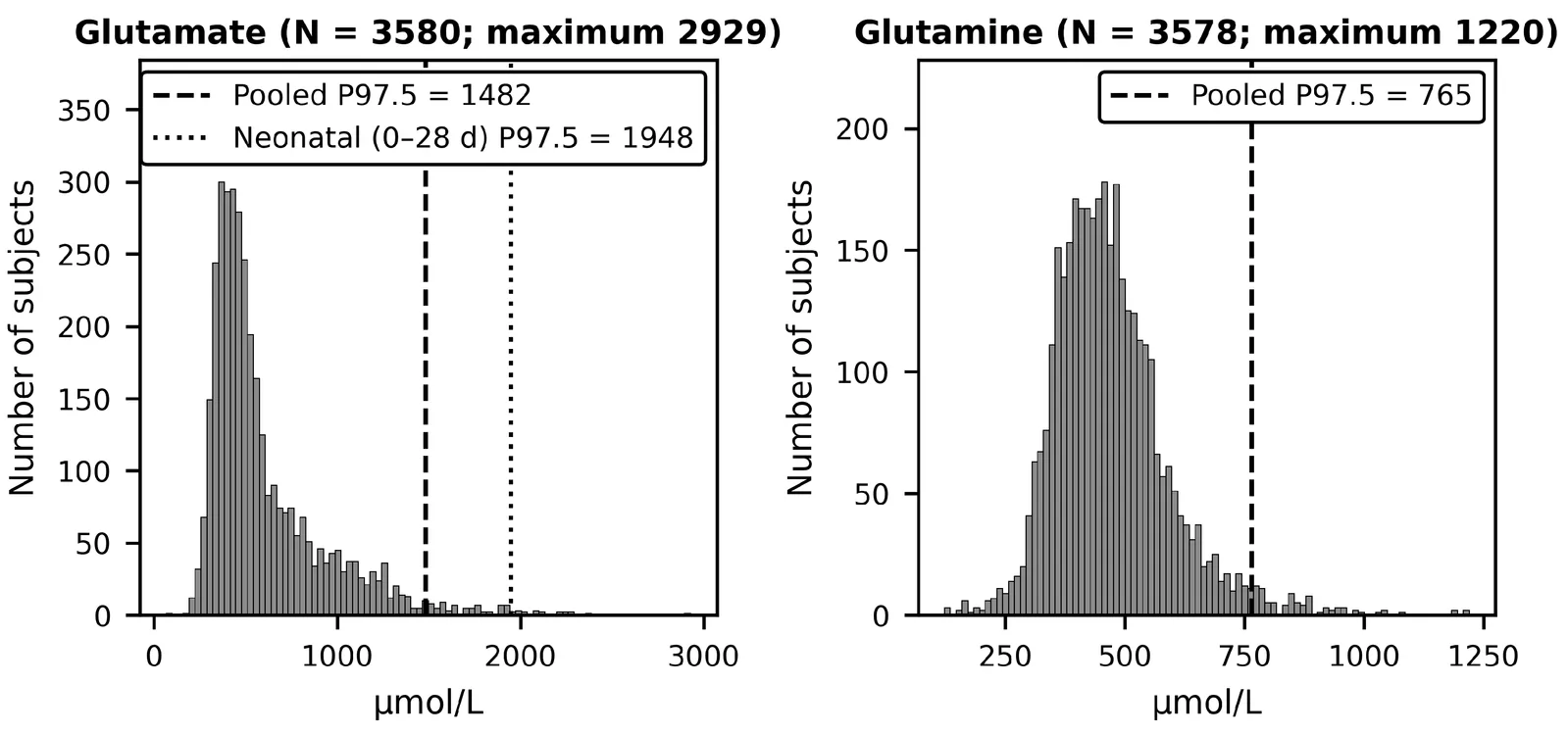
Glutamate and glutamine distributions on the adopted cohort with naive and outlier-controlled 97.5th-percentile limits.

Glutamate and glutamine are non-specific analytes that the LIS reference range over-flags. Glutamate was flagged high in about 19% of records. In the neonatal (0–28-day) stratum, its 97.5th-percentile upper limit on the adopted cohort was 1947.6 μmol/L (Supplementary Table S1). This limit is reproducible rather than an outlier artefact: robust trimming (3 × IQR on the log scale) left it unchanged, because the trimming cap (4328 μmol/L) lay above the observed maximum (2929 μmol/L). Its magnitude reflects two factors. The first is the correction of over-flagging: exempting the two non-specific analytes approximately doubled the eligible cohort (1842 to 3589 subjects), and, pooled across all ages, the glutamate 97.5th percentile was 886 μmol/L under strict exclusion versus 1482 μmol/L on the adopted cohort. The second is the specimen matrix: DBS results are whole-blood concentrations, and glutamate belongs to the group of anionic amino acids that erythrocytes actively concentrate, reaching high erythrocyte-to-plasma ratios, whereas glutamine is carried by a non-concentrating transport system [23]. Whole-blood glutamate is therefore expected to exceed the corresponding plasma concentration, and DBS values for this analyte are not interchangeable with plasma-derived reference data. Consistent with this distinction, glutamine was flagged low in about 32% of records and has a well-behaved neonatal upper limit of 889 μmol/L, so the high neonatal limit is specific to glutamate. Glutamine is also the least stable of the amino acids measurable in stored DBS [24], which plausibly contributes to its high low-flag rate. Both distributions and their naive and outlier-controlled limits are shown in Figure 8. We report both analytes with outlier-controlled limits and use neither as the basis for a fixed cut-off.

### 3.6. Cross-Validation by an Independent Indirect Method

Reference limits estimated with the canonical refineR package from the full, unselected distribution agreed with the flag-based CLSI estimates within ±30% in 91% of key-marker limits (within ±20% in 80%; median relative difference 9.6%; Table 5; full per-stratum comparison in Table S3). The clinically decisive upper limits agreed within a narrow band (median absolute difference 12.1%, maximum 31.8%; 36 of 37 within ±30%, the sole exception being C5 at 29 days–1 year). The divergences beyond 30% were otherwise confined to the lower limits of near-zero analytes (arginine and neonatal C3, C16 and C18; six strata), where a difference of hundredths of a μmol/L yields a large relative value (lower-limit median 8.0%, maximum 85.4%). The ±30% criterion is therefore an upper bound set by these near-zero lower limits rather than by the clinically used upper limits. Because the flag-independent estimator uses none of the selection flags, this convergence is direct evidence that flag-based selection did not materially bias the intervals. An in-house refineR-equivalent gave broadly similar results but occasionally over-estimated an upper limit (e.g., neonatal phenylalanine), reinforcing preference for the canonical package.

### 3.7. External Comparison

**Figure 9:**
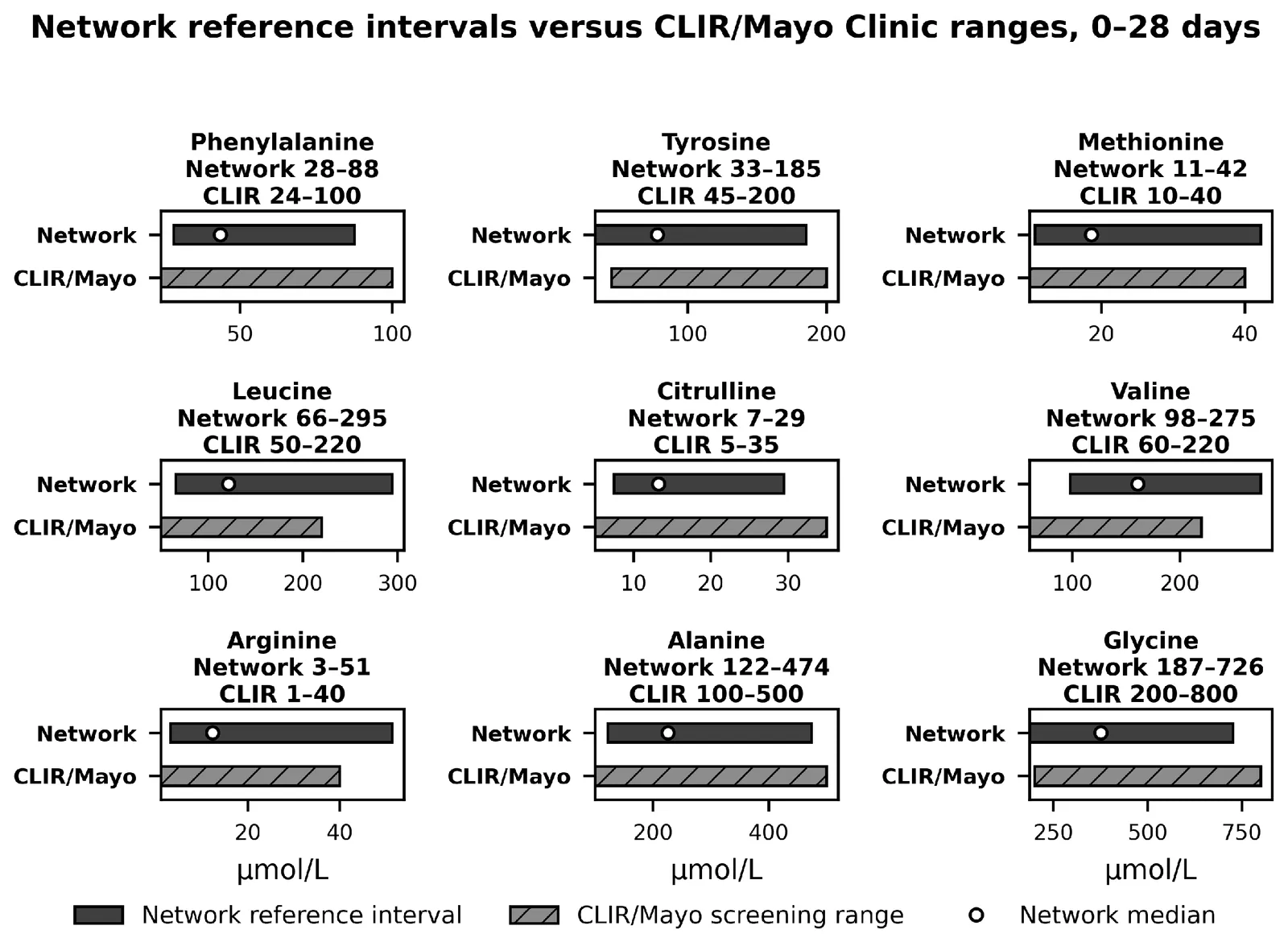
Network neonatal (0–28 d) reference intervals compared with CLIR/Mayo ranges, which are screening cut-offs rather than population reference intervals.

All nine key markers at 0–28 days were comparable with CLIR/Mayo post-analytical ranges (Table 6 and Figure 9); these ranges are presented as clinical context, not as external validation, because they are screening cut-offs rather than population reference intervals. Where the network upper limit exceeded a CLIR range (e.g., leucine, valine), this reflects that distinction. Versus the Turkish study, most limits differed by <25%, and neonatal glycine lower limits were near-identical (186.6 vs. 181 μmol/L).

### 3.8. Inter-Site Comparison

**Figure 10:**
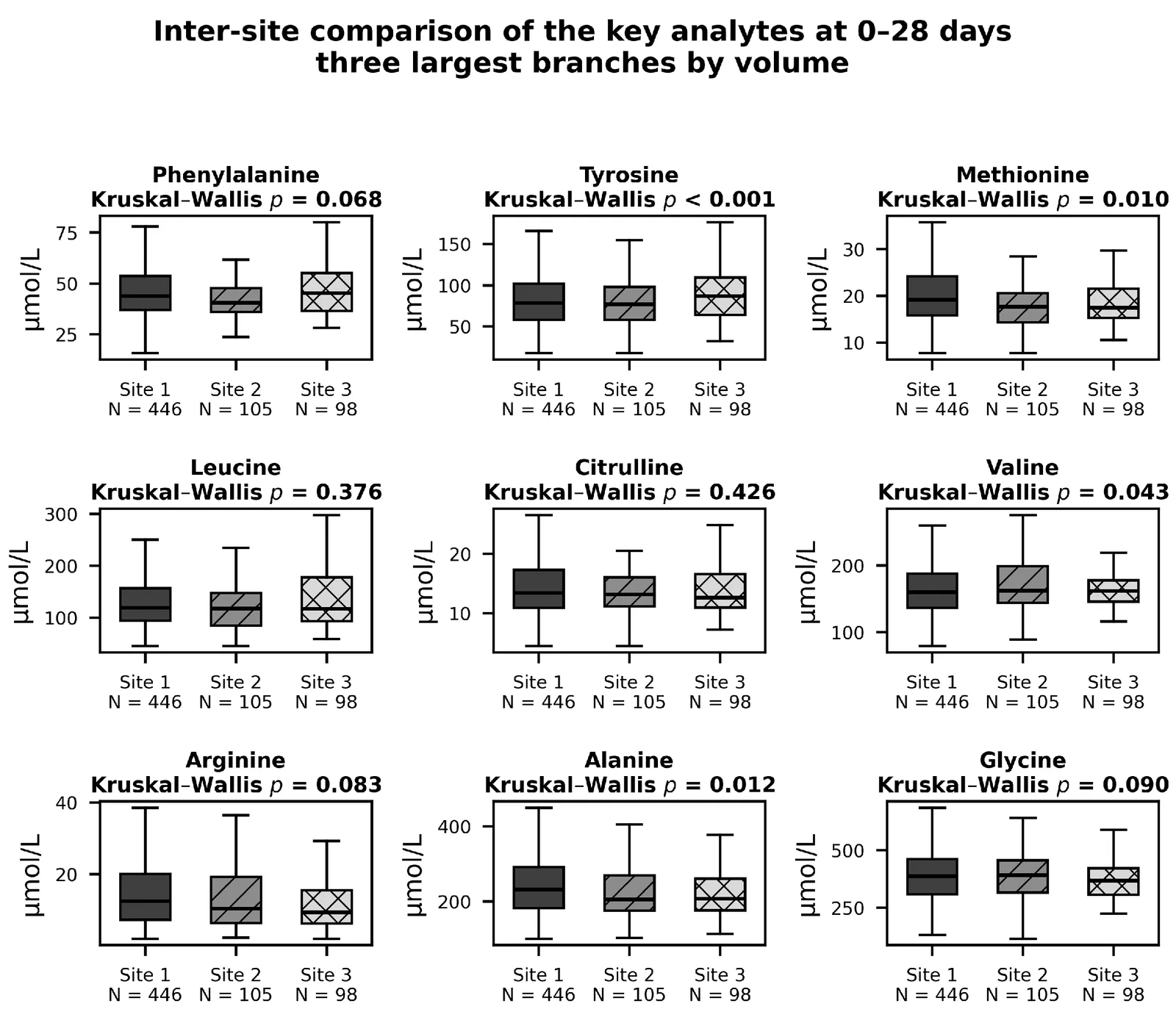
Inter-site variation of neonatal (0–28 d) concentrations of the key markers across the three largest branches by volume (Site 1, Astana; Site 2, Ust-Kamenogorsk; Site 3, Shymkent). Boxes span the interquartile range, the horizontal line marks the median and the whiskers extend to 1.5 × IQR; individual points beyond the whiskers are omitted for legibility.

Across 27 inter-site Kruskal–Wallis comparisons (9 key markers across the 3 best-populated strata among the 4 largest branches), 13 reached nominal significance and 6 survived Bonferroni correction (α=0.00185): phenylalanine, citrulline, valine and methionine at 29 days–1 year, tyrosine at 0–28 days and citrulline at 1–4 years (Figure 10; the complete per-analyte results are given in Table S2). The larger count relative to the strict cohort reflects the greater statistical power of the expanded cohort, not large absolute differences, as between-site median differences were small relative to interval width. Unified network-wide RIs therefore remain appropriate, while these analytes, and phenylalanine in particular, are prioritised for prospective site-level harmonisation monitoring.

### 3.9. Sex Differences

**Figure 11:**
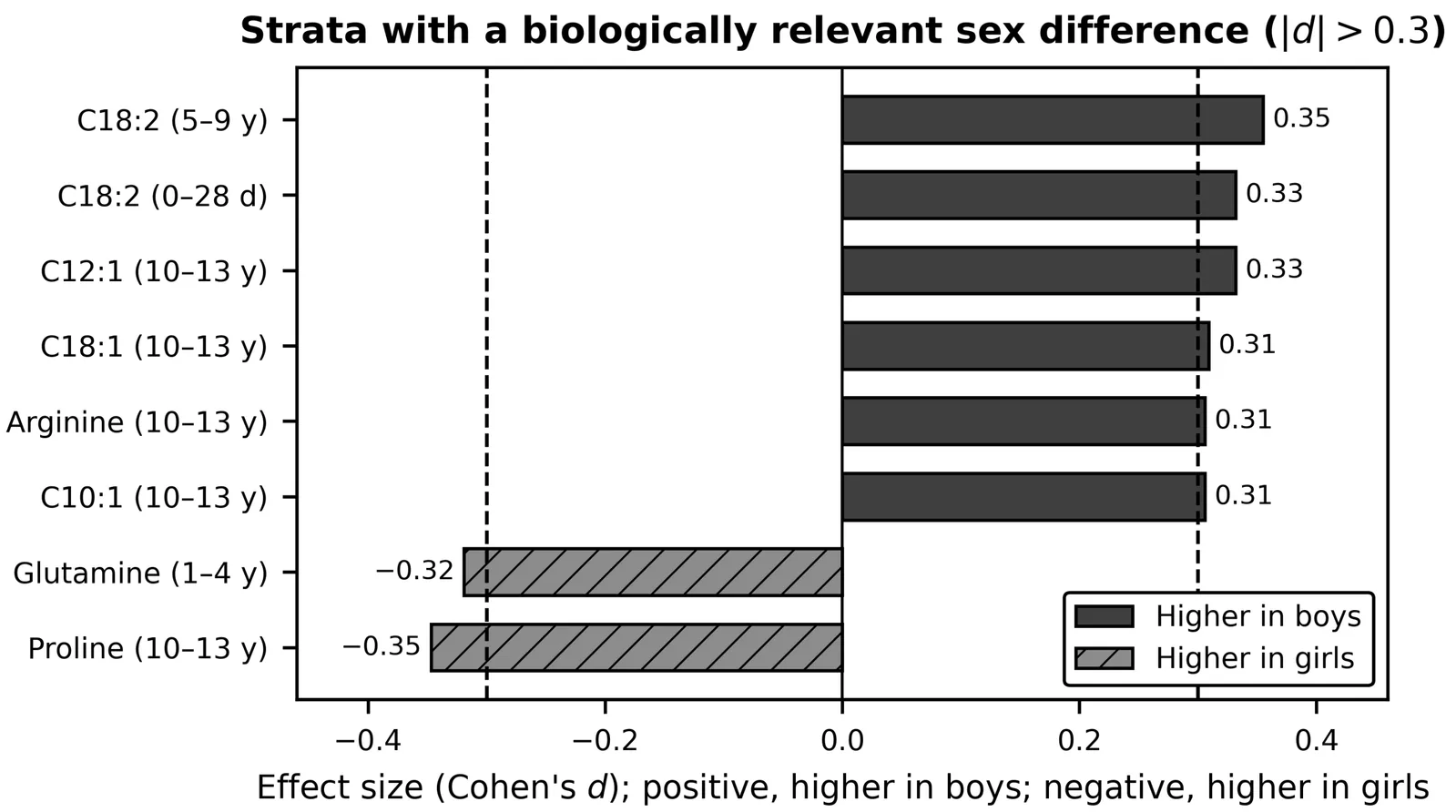
Strata with a biologically relevant sex difference (|d|>0.3); dashed lines mark ±0.3.

Biologically relevant sex differences (|d|>0.3) occurred in only 8 of 250 sufficiently large strata (3.2%), all of modest magnitude (|d|≤0.36) and concentrated in older children (Figure 11; complete per-stratum results in Table S4); the remainder showed no relevant dimorphism, so sex partitioning was not required for the panel as a whole.

### 3.10. Distributional Diagnostics and Clinical-Limit Proximity

No stratum was Gaussian-normal (about 69% non-normal, 31% log-normal), validating the non-parametric approach; Box–Cox was a sensitivity check only; extreme outlier (3 × IQR) contamination was low (mean 0.77%). A rolling CUSUM analysis detected measurable temporal fluctuation in a majority of windows, so results were not perfectly stationary over 2022–2026 (addressed as a limitation). The valine 97.5th-percentile upper limit exceeded the 250 μmol/L maple syrup urine disease decision limit at 0–28 days (275.3 μmol/L, +10.1%), and leucine approached its 300 μmol/L limit (294.5 μmol/L).

## 4. Discussion

To our knowledge, this is the first study to establish age- and sex-specific paediatric MS/MS reference intervals for amino acids and acylcarnitines in a Kazakhstani population. By exploiting more than 687,000 routine results across a national, 21-branch network, the indirect approach yielded strata large enough to apply the full CLSI EP28-A3c method in the great majority of cases, a coverage rarely achievable by direct sampling of healthy neonates and children [4,7,8].

These intervals also fill a specific geographical gap. Large population- or nation-scale MS/MS reference efforts have been reported for other regions, most notably a nationwide Chinese study spanning millions of neonates [25] and an indirect, laboratory-information-system-based Turkish paediatric study [21]. To our knowledge, none exist for any Central Asian population. Establishing locally derived intervals matters because amino-acid and acylcarnitine concentrations are influenced by genetic background, feeding practice and sampling logistics [7]. Our values are broadly concordant with these international datasets while differing in clinically relevant tails (e.g., neonatal valine), underscoring why transference of external limits should be verified rather than assumed.

A central strength is the dual, independent estimation of every key interval. Conventional flag-based selection and the canonical refineR package applied to the unfiltered distribution converged in the large majority of strata [17]. Because the two estimators rest on different assumptions, their agreement is evidence that the selection rule did not materially bias the intervals; this cross-check is absent from most comparable indirect studies, including the otherwise closely matched Turkish study [21]. Indirect, data-mining approaches are increasingly endorsed precisely for settings such as neonatal and paediatric testing, where assembling a sufficiently large sample of demonstrably healthy reference subjects is impractical [4,26].

The analysis also yielded a practical methodological lesson: a single out-of-range flag on a non-specific, over-flagged analyte such as glutamate or glutamine should not exclude a subject from a healthy reference cohort. Correcting for this over-flagging roughly doubled the eligible cohort and the glutamate upper limit; we interpret this cautiously as evidence that the local flag limit for these non-specific analytes is set conservatively, rather than as a definitive statement about assay calibration. For glutamate, the whole-blood matrix of DBSs contributes as well: because erythrocytes concentrate anionic amino acids, DBS glutamate necessarily exceeds the plasma concentration and cannot be interpreted against plasma-derived limits [23]. Locally derived DBS intervals, reported here for both analytes, are therefore the only sound basis for flagging them, and we retain them in the panel for that reason.

Two findings are clinically actionable. Several key markers differed significantly between sites even after Bonferroni correction on the expanded cohort; although the absolute between-site median differences were small relative to interval width, these analytes warrant harmonisation monitoring, particularly phenylalanine, which is pivotal for phenylketonuria detection. The upper-normal valine limit exceeded its 250 μmol/L decision limit at 0–28 days (275.3 μmol/L) and leucine approached its 300 μmol/L limit, so interpretation should not rely on a single upper percentile but should incorporate confirmatory and ratio-based post-analytical logic [19,20,27]. Post-analytical, multi-variate and ratio-based tools (e.g., the CLIR framework) and second-tier testing were developed specifically to resolve this tension and are preferable to any single percentile in such markers [19,20]. Several acylcarnitines and ratio-based indicators require different threshold logic; the per-stratum limits and computation status for every quantitative analyte of the panel are reported with that caveat in Table S1.

Beyond their analytical role, locally derived intervals let the network replace heterogeneous, vendor-supplied limits with a single, internally cross-checked set, reducing avoidable repeat testing and inconsistent flagging between branches. The complete adopted reference-interval table (Supplementary Table S1) is intended to serve as the implementation reference for the network’s laboratories.

This study has limitations. The indirect method assumes that the analysed population is predominantly healthy and cannot fully exclude subclinical conditions; it reflects a single platform and network, so transference to other settings should be verified [28]; and some older-age strata remained below the full-method threshold. Although specimen quality, transport and storage were controlled and documented per CLSI NBS01 (Section 2), the consolidated LIS holds no individual-level clinical outcome, feeding, gestational-age, birthweight, or nursery data and no per-subject pre-analytical timing; subclinical conditions therefore cannot be fully excluded, and neonatal sub-classification is by post-natal age only. The CUSUM analysis indicated non-negligible temporal fluctuation, so pooling of 2022–2026 results is a pragmatic simplification and the network should monitor for analytical drift. Acylcarnitine and ratio-based limits warrant dedicated evaluation.

## 5. Conclusions

We provide the first locally derived and internally cross-checked paediatric MS/MS reference intervals for amino acids and acylcarnitines in a Kazakhstani population and, to our knowledge, in any Central Asian population, derived from a large, multi-branch national dataset using a reproducible indirect method. We recommend prospective, site-level verification of these limits before they replace existing vendor-supplied limits; once verified, they can be adopted network-wide, together with inter-site harmonisation monitoring of the markers identified above and ratio-based, second-tier interpretation of valine near its diagnostic decision limit rather than reliance on a single percentile. Methodologically, the dual flag-based/flag-independent estimation framework offers a practical template for indirect reference-interval studies, and the over-flagging lesson may inform other indirect studies. Because reference intervals are not static, the network should treat these limits as a baseline to be verified prospectively and re-derived periodically [5,28]; the temporal fluctuation detected here reinforces that need.

## Figures and Tables

**Table 1:** Effect of the cohort-selection rule on the number of conditionally healthy subjects (fresh extract, data freeze 20 July 2026).

Selection Scenario	Conditionally Healthy
Strict (exclude on any out-of-range flag)	1842
Ignore glutamate only	2466
Ignore glutamine only	2835
Ignore glutamate + glutamine (adopted)	3589
Total unique patients	4198

**Table 2:** Age-specific reference intervals (μmol/L) for the nine key amino-acid markers (adopted cohort). EP28, full CLSI EP28 (N≥120); CI, 90% bootstrap confidence interval of the upper limit.

Analyte	Age	*N*	Method	2.5th	Median	97.5th	97.5th CI
Phenylalanine	0–28 d	777	CLSI EP28	28.2	43.6	87.5	81.0–95.7
Tyrosine	0–28 d	777	CLSI EP28	33.2	78.1	185.3	168.2–189.0
Methionine	0–28 d	777	CLSI EP28	10.8	18.7	42.2	36.7–44.5
Leucine	0–28 d	777	CLSI EP28	65.8	121.6	294.5	271.9–305.1
Citrulline	0–28 d	777	CLSI EP28	7.4	13.2	29.5	26.3–30.5
Citrulline	1–4 y	896	CLSI EP28	10.6	22.6	39.0	36.7–40.1
Valine	0–28 d	777	CLSI EP28	98.0	160.7	275.3	248.2–287.2
Arginine	0–28 d	777	CLSI EP28	3.0	12.3	51.5	43.2–57.5
Alanine	0–28 d	777	CLSI EP28	121.6	226.2	474.3	437.9–508.8
Glycine	0–28 d	777	CLSI EP28	186.6	375.8	726.1	684.4–775.4

**Table 3:** Reference intervals (μmol/L) for the principal acylcarnitines at 0–28 days and 29 days–1 year (adopted cohort); full set for all 36 acylcarnitines in Supplementary Table S1.

Acylcarnitine	Age	*N*	2.5th	Median	97.5th
C0	0–28 d	777	12.126	22.914	57.051
C0	29 d–1 y	707	15.766	35.611	71.549
C2	0–28 d	777	7.072	16.524	41.777
C2	29 d–1 y	707	6.832	16.630	40.086
C3	0–28 d	777	0.650	2.103	4.937
C3	29 d–1 y	707	0.674	1.743	4.750
C5	0–28 d	777	0.078	0.182	0.527
C5	29 d–1 y	707	0.069	0.145	0.433
C8	0–28 d	777	0.028	0.060	0.130
C8	29 d–1 y	707	0.024	0.055	0.143
C10	0–28 d	777	0.028	0.066	0.152
C10	29 d–1 y	707	0.027	0.065	0.183
C14	0–28 d	777	0.046	0.179	0.367
C14	29 d–1 y	707	0.038	0.108	0.298
C16	0–28 d	777	0.423	2.731	5.672
C16	29 d–1 y	707	0.328	0.988	2.532
C18	0–28 d	777	0.260	0.773	1.480
C18	29 d–1 y	707	0.194	0.484	1.048
C18:1	0–28 d	777	0.475	1.262	2.370
C18:1	29 d–1 y	707	0.350	1.055	2.657

**Table 4:** Reference intervals (μmol/L) within the first month for key markers, split into 0–7/8–14/15–28 days (adopted cohort). full, full CLSI EP28 computation (N≥120); prov., provisional (40≤N<120).

Analyte	Stratum	*N*	2.5th	Median	97.5th	Status
C3	0–7 d	522	1.199	2.449	5.033	full
C3	8–14 d	138	0.614	1.294	3.309	full
C3	15–28 d	117	0.494	1.276	3.440	prov.
C16	0–7 d	522	1.209	3.285	6.050	full
C16	8–14 d	138	0.724	1.753	4.450	full
C16	15–28 d	117	0.340	0.811	2.314	prov.
C5	0–7 d	522	0.074	0.172	0.472	full
C5	8–14 d	138	0.092	0.232	0.594	full
C5	15–28 d	117	0.103	0.214	0.782	prov.
C0	0–7 d	522	12.695	22.487	47.648	full
C0	8–14 d	138	11.423	22.519	57.013	full
C0	15–28 d	117	12.153	24.769	65.484	prov.

**Table 5:** Concordance of reference-limit estimates: direct flag-based CLSI EP28 vs. canonical refineR (full cohort, no flag-based selection) for the key markers.

Concordance Metric	Value
Compared limits (9 amino acids × 3 strata + 5 acylcarnitines × 2 strata)	74
Median relative difference, canonical refineR vs. direct	9.6%
Limits within ±20%	80%
Limits within ±30%	91%
Largest upper-limit divergences	C5 29 d–1 y, glycine 29 d–1 y, methionine 1–4 y
Largest lower-limit divergences	near-zero acylcarnitines (C16) and arginine

Full per-stratum three-way table in Supplementary Table S3.

**Table 6:** Neonatal (0–28 days) reference intervals vs. CLIR/Mayo Clinic ranges (μmol/L). CLIR/Mayo ranges are screening cut-offs, not population RIs.

Analyte	*N*	2.5th	Median	97.5th	CLIR/Mayo
Phenylalanine	777	28.2	43.6	87.5	24–100
Tyrosine	777	33.2	78.1	185.3	45–200
Methionine	777	10.8	18.7	42.2	10–40
Leucine	777	65.8	121.6	294.5	50–220
Citrulline	777	7.4	13.2	29.5	5–35
Valine	777	98.0	160.7	275.3	60–220
Arginine	777	3.0	12.3	51.5	1–40
Alanine	777	121.6	226.2	474.3	100–500
Glycine	777	186.6	375.8	726.1	200–800

## Data Availability

The de-identified summary statistics (reference intervals and supporting tables) are available within the article and its Supplementary Materials. The extraction and analysis code (SQL, Python and the canonical refineR R script) and the de-identified aggregate results are openly available on Zenodo at https://doi.org/10.5281/zenodo.21508934 (code under the MIT License; derived data under CC-BY-4.0). Raw individual-level laboratory records are not publicly available owing to privacy and institutional restrictions.

## Supplementary Materials

The following supporting information can be downloaded at: https://www.mdpi.com/article/10.3390/diagnostics16172766/s1, Table S1: full per-stratum reference intervals for all analytes, including all 36 acylcarnitines and the fractional neonatal strata (the version intended for adoption in the network); Table S2: complete Kruskal–Wallis inter-site results; Table S3: three-way comparison of direct, in-house and canonical refineR limits; Table S4: complete per-stratum sex-difference results (Cohen’s *d*); Methods S1: study parameters and computational settings (data freeze, cohort-selection rule, reference-interval method, bootstrap settings, random seed, refineR configuration, software versions and the data-privacy statement). All extraction and analysis code (SQL, Python, R; Transact-SQL, Python 3.14.6 and R 4.6.1) and the de-identified aggregate results are openly available on Zenodo at https://doi.org/10.5281/zenodo.21508934 (see Data Availability Statement).

## References

[1] Chace D.H., Kalas T.A., Naylor E.W. (2003). Use of tandem mass spectrometry for multianalyte screening of dried blood specimens from newborns. Clin. Chem..

[2] La Marca G. (2014). Mass spectrometry in clinical chemistry: The case of newborn screening. J. Pharm. Biomed. Anal..

[3] Clinical and Laboratory Standards Institute (2010). Defining, Establishing, and Verifying Reference Intervals in the Clinical Laboratory; Approved Guideline—Third Edition (EP28-A3c).

[4] Jones G.R., Haeckel R., Loh T.P., Sikaris K., Streichert T., Katayev A., Barth J.H., Ozarda Y. (2019). IFCC Committee on Reference Intervals and Decision Limits Indirect methods for reference interval determination—Review and recommendations. Clin. Chem. Lab. Med..

[5] Ozarda Y., Higgins V., Adeli K. (2019). Verification of reference intervals in routine clinical laboratories: Practical challenges and recommendations. Clin. Chem. Lab. Med..

[6] Henny J., Vassault A., Boursier G., Vukasovic I., Brguljan P.M., Lohmander M., Ghita I., Andreu F.A.B., Kroupis C., Sprongl L. (2016). Recommendation for the review of biological reference intervals in medical laboratories. Clin. Chem. Lab. Med..

[7] Colantonio D.A., Kyriakopoulou L., Chan M.K., Daly C.H., Brinc D., Venner A.A., Pasic M.D., Armbruster D., Adeli K. (2012). Closing the gaps in pediatric laboratory reference intervals: A CALIPER database of 40 biochemical markers. Clin. Chem..

[8] Adeli K., Higgins V., Trajcevski K., White-Al Habeeb N. (2017). The Canadian laboratory initiative on pediatric reference intervals: A CALIPER white paper. Crit. Rev. Clin. Lab. Sci..

[9] Tahmasebi H., Higgins V., Fung A.W.S., Truong D., White-Al Habeeb N.M.A., Adeli K. (2017). Pediatric reference intervals for biochemical markers: Gaps and challenges, recent national initiatives and future perspectives. EJIFCC.

[10] Pavlov I.Y., Wilson A.R., Delgado J.C. (2012). Reference interval computation: Which method (not) to choose?. Clin. Chim. Acta.

[11] Ozarda Y. (2016). Reference intervals: Current status, recent developments and future considerations. Biochem. Med..

[12] Zharmakhanova G., Kononets V., Balmagambetova S., Syrlybayeva L., Nurbaulina E., Zhussupova Z., Sakhanova S., Ayaganov D., Kim S., Zhumalina A. (2024). Selective screening for inborn errors of metabolism using tandem mass spectrometry in West Kazakhstan children: Study protocol. Front. Genet..

[13] Borovecki A., Mlinaric A., Horvat M., Šupak Smolčić V. (2018). Informed consent and ethics committee approval in laboratory medicine. Biochem. Med..

[14] Clinical and Laboratory Standards Institute (2021). Dried Blood Spot Specimen Collection for Newborn Screening (NBS01).

[15] Dietzen D.J., Rinaldo P., Whitley R.J., Rhead W.J., Hannon W.H., Garg U.C., Lo S.F., Bennett M.J. (2009). National Academy of Clinical Biochemistry laboratory medicine practice guidelines: Follow-up testing for metabolic disease identified by expanded newborn screening using tandem mass spectrometry; executive summary. Clin. Chem..

[16] Clinical and Laboratory Standards Institute (2014). Liquid Chromatography-Mass Spectrometry Methods; Approved Guideline (C62-A).

[17] Ammer T., Schützenmeister A., Prokosch H.U., Rauh M., Rank C.M., Zierk J. (2021). refineR: A novel algorithm for reference interval estimation from real-world data. Sci. Rep..

[18] McHugh D., Cameron C.A., Abdenur J.E., Abdulrahman M., Adair O., Al Nuaimi S.A., Åhlman H., Allen J.J., Antonozzi I., Archer S. (2011). Clinical validation of cutoff target ranges in newborn screening of metabolic disorders by tandem mass spectrometry. Genet. Med..

[19] Marquardt G., Currier R., McHugh D., Gavrilov D., Magera M.J., Matern D., Oglesbee D., Raymond K., Rinaldo P., Smith E.H. (2012). Enhanced interpretation of newborn screening results without analyte cutoff values. Genet. Med..

[20] Hall P.L., Marquardt G., McHugh D., Currier R.J., Tang H., Stoway S.D., Rinaldo P. (2014). Postanalytical tools improve performance of newborn screening by tandem mass spectrometry. Genet. Med..

[21] Madenci Ö.Ç., Erdin S., Kestane A., Kutnu M. (2023). Establishment of age- and gender-specific reference intervals for amino acids and acylcarnitines by tandem mass spectrometry in Turkish paediatric population. Biochem. Med..

[22] Cohen J. (1988). Statistical Power Analysis for the Behavioral Sciences.

[23] Hagenfeldt L., Arvidsson A. (1980). The distribution of amino acids between plasma and erythrocytes. Clin. Chim. Acta.

[24] Dijkstra A.M., de Blaauw P., van Rijt W.J., Renting H., Maatman R.G., van Spronsen F.J., Maase R.E., Schielen P.C., Derks T.G., Heiner-Fokkema M.R. (2023). Important lessons on long-term stability of amino acids in stored dried blood spots. Int. J. Neonatal Screen..

[25] He F., Yang R., Huang X., Tian Y., Pei X., Bohn M.K., Zou L., Wang Y., Li H., Wang T. (2021). Reference standards for newborn screening of metabolic disorders by tandem mass spectrometry: A nationwide study on millions of Chinese neonatal populations. Front. Mol. Biosci..

[26] Farrell C.J., Nguyen L. (2019). Indirect reference intervals: Harnessing the power of stored laboratory data. Clin. Biochem. Rev..

[27] Strauss K.A., Carson V.J., Soltys K., Young M.E., Bowser L.E., Puffenberger E.G., Brigatti K.W., Williams K.B., Robinson D.L., Hendrickson C. (2020). Branched-chain *α*-ketoacid dehydrogenase deficiency (maple syrup urine disease): Treatment, biomarkers, and outcomes. Mol. Genet. Metab..

[28] Tate J.R., Yen T., Jones G.R.D. (2015). Transference and validation of reference intervals. Clin. Chem..

